# The vulnerability of migrants living in large urban areas to COVID-19: Exacerbators and mitigators

**DOI:** 10.1093/eurpub/ckac130.168

**Published:** 2022-10-25

**Authors:** L Hitch, K Cravero, D Masoud, M Moujabber, LA Hobbs

**Affiliations:** Center for Immigrant, Refugee & Global Health, CUNY Graduate School of Public Health, New York, USA

## Abstract

**Background:**

Even though large urban areas have been researched in the context of COVID-19, evidence on how COVID-19 impacted migrants -a particularly vulnerable group- in these settings is still limited.

Objective: To explore exacerbating and mitigating factors of large urban areas on migrants’ vulnerabilities during the COVID-19 pandemic.

**Methods:**

We conducted a systematic review of peer-reviewed literature published between 2020-2022 and focused on migrants (foreign-born individuals who have not been naturalized in the host country, regardless of immigration status) in urban areas with a population >500.000. After screening 880 studies, 29 studies were included and categorized within the following thematic framework: 1) Underlying structural inequities, 2) governance and economic structure, 3) urban design, and 4) engagement of civil society organizations (CSO).

**Results:**

Exacerbating factors include pre-existing inequities (e.g., unemployment, financial precarity, and barriers to healthcare access), exclusionary government responses (e.g., relief funds or unemployment benefits), and residential segregation. Mitigating factors include the engagement of CSOs and the implementation of innovative governance strategies such as e-governance and use of teleservices. Recommendations: We recommend increased attention to pre-existing social inequities faced by migrants, inclusive governance strategies, and partnerships between government and CSOs to improve the design and delivery of services to migrants in large urban areas. More research is needed on how urban design can be utilized to mitigate the COVID-19 impacts on migrant communities.

**Conclusions:**

The factors identified in this systematic review should be considered as part of migrant-inclusive emergency preparedness to address the disproportionate impact of similar public health crises on migrant communities.

**Key messages:**

- Pre-existing inequities, exclusionary governance strategies, and residential segregation led to a disproportionate impact of COVID-19 on migrants living in large urban areas.
- Mitigators of cities on the COVID-19 impacts among migrants include the engagement of civil society, implementation of innovative governance strategies, and use of teleservices in these settings.

